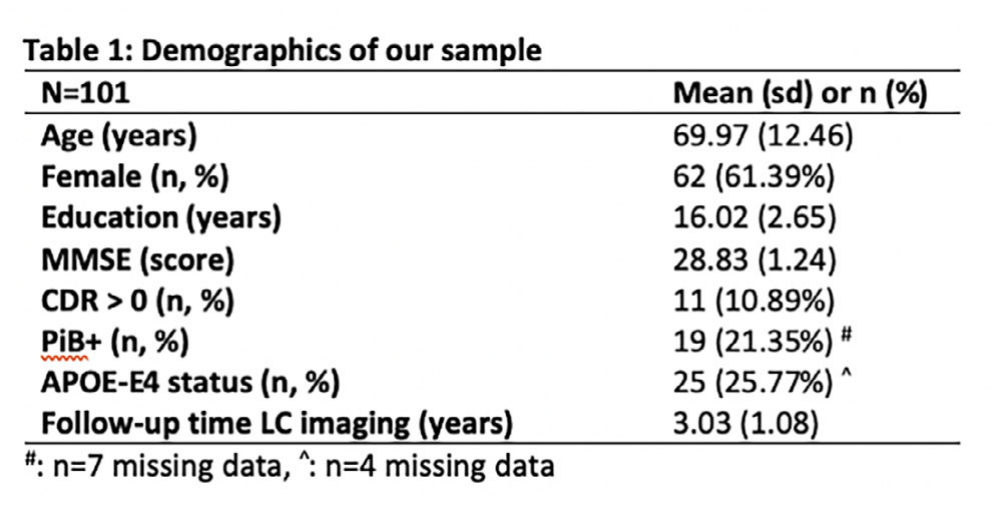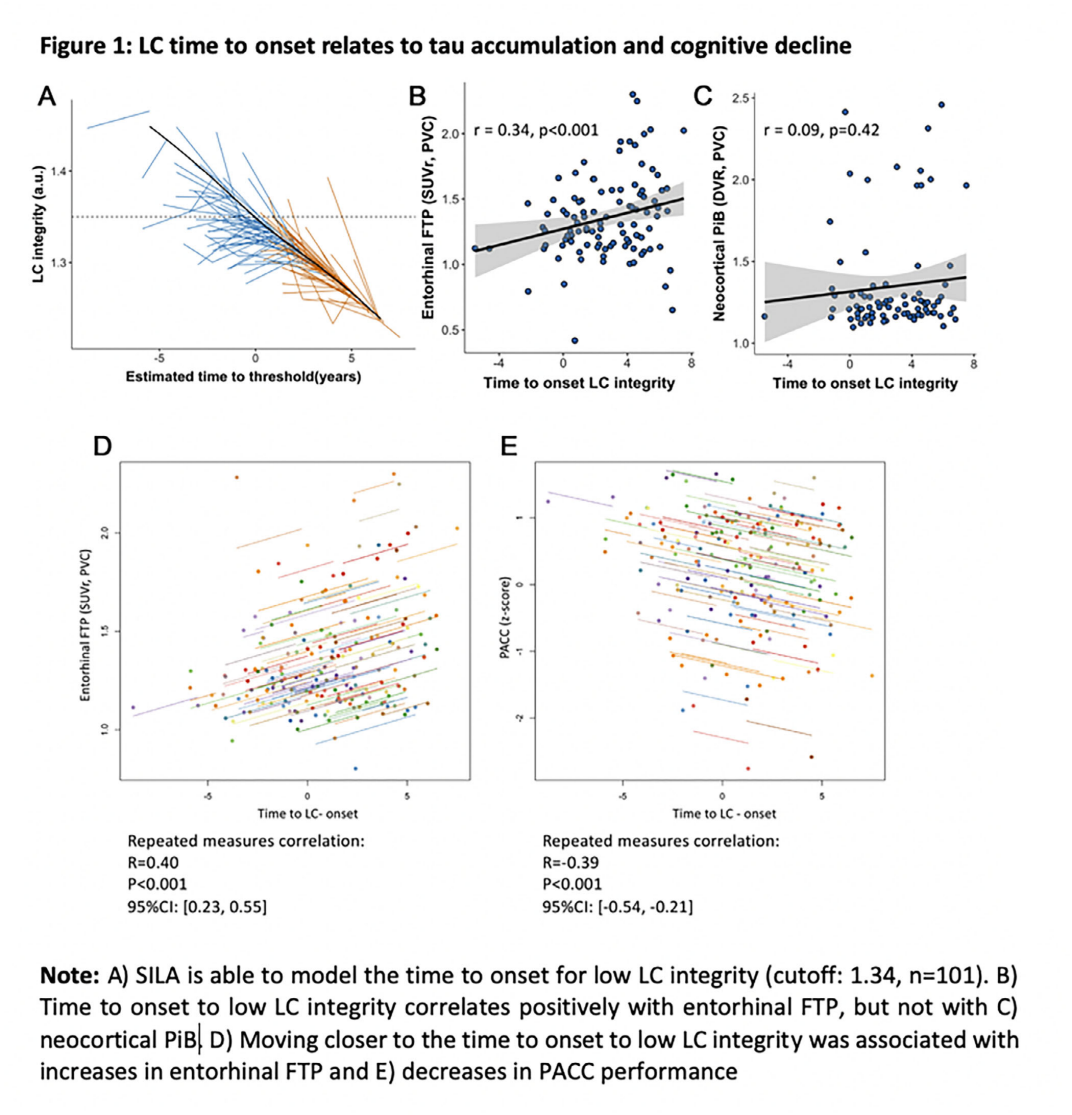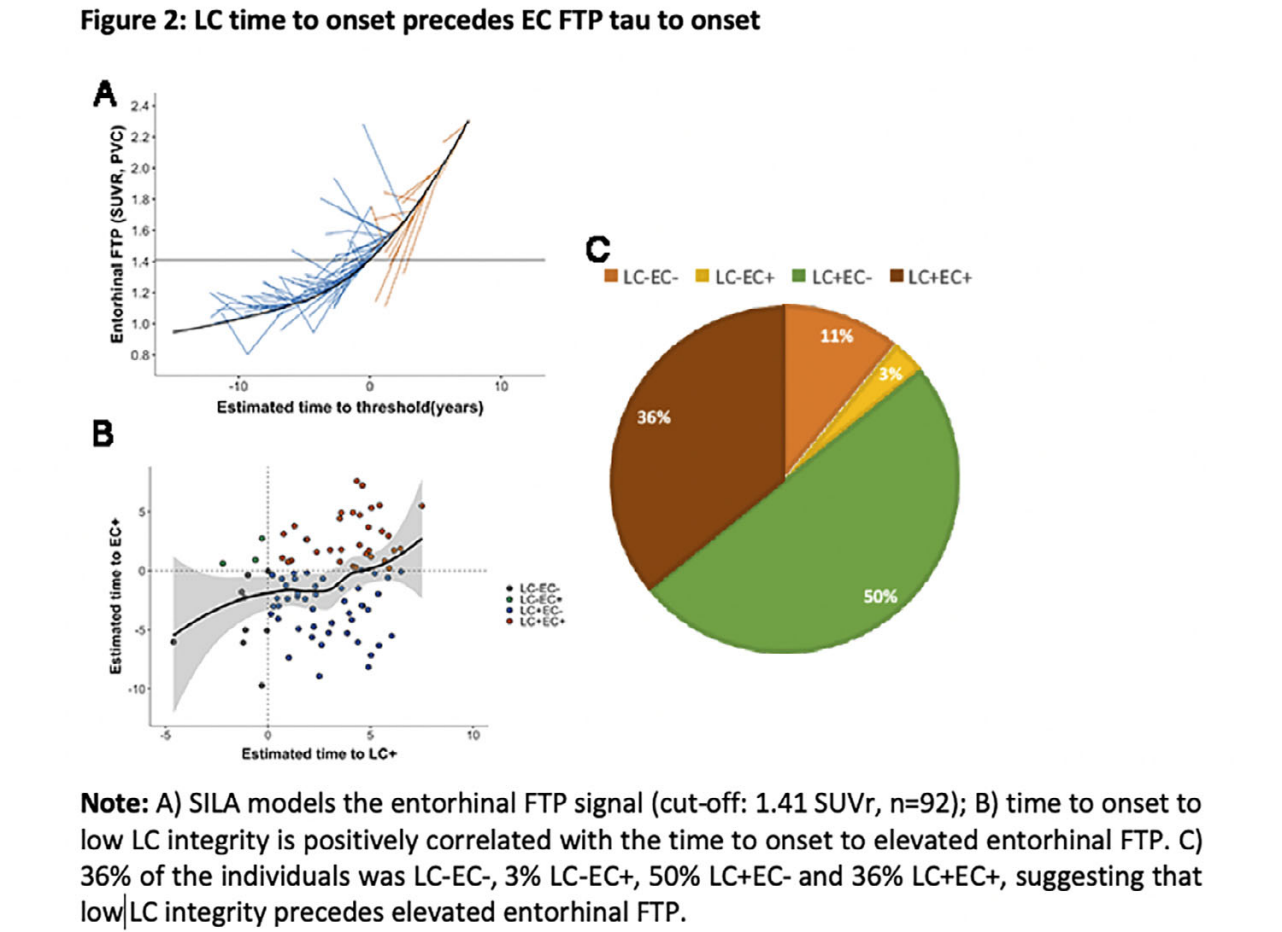# Linking the estimated time of onset of low locus coeruleus integrity to the estimated time of onset of elevated entorhinal tau

**DOI:** 10.1002/alz.091078

**Published:** 2025-01-09

**Authors:** Heidi I.L. Jacobs, Tobey J. Betthauser, Alex Becker, Joost M. Riphagen, Kathryn V Papp, Julie C Price, Dorene M Rentz, Reisa A Sperling, Keith A Johnson

**Affiliations:** ^1^ Massachusetts General Hospital, Harvard Medical School, Boston, MA USA; ^2^ Gordon Center for Medical Imaging, Massachusetts General Hospital, Harvard Medical School, Boston, MA USA; ^3^ Alzheimer’s Disease Research Center, University of Wisconsin‐Madison, Madison, WI USA; ^4^ Massachusetts General Hospital, Brigham and Women's Hospital, Harvard Medical School, Boston, MA USA; ^5^ Massachusetts General Hospital, Boston, MA USA; ^6^ Brigham and Women's Hospital, Harvard Medical School, Boston, MA USA; ^7^ Harvard Medical School, Boston, MA USA; ^8^ Center for Alzheimer’s Research and Treatment, Brigham and Women’s Hospital, Massachusetts General Hospital, Harvard Medical School, Boston, MA USA

## Abstract

**Background:**

Estimating the time course of locus coeruleus integrity changes is important for a better understanding of the pathophysiological cascade and for identifying the optimal window of opportunity for prevention trials. We used samples iterative local approximation (SILA) to determine the individual estimated time of onset of low LC integrity and related this to early cortical tau deposition and cognitive decline.

**Methods:**

101 individuals from the Harvard Aging Brain Study+ with longitudinal 3T MRI‐LC imaging, 18F‐Flortaucipir (FTP)‐PET imaging (n=92 with longitudinal data), longitudinal cognitive assessments and baseline PiB‐PET imaging were included (mean age: 69.97 years (SD: 12.46); 62 females; Table 1). LC intensity was derived by normalizing the LC to the pontine reference and averaging 5‐voxel‐clusters with the highest intensities (GMM‐based cut‐off: <1.34 = LC+). FTP‐burden was quantified in the entorhinal cortex (cerebellar grey reference region, partial volume corrected, GMM‐based cut‐off: >1.41 SUVr = EC+). SILA was used to model trajectories of LC intensity and EC FTP separately. Time to onset of LC+ was correlated with baseline entorhinal tau and neocortical amyloid. Repeated measures correlations related the time to onset to LC+ to longitudinal entorhinal FTP and longitudinal PACC scores. Time to onset to LC+ and elevated EC+ were correlated to each other.

**Results:**

75.25% (n=76) of the individuals were considered LC+ (Figure 1A) and 40.22% (n=37) was EC+ (Figure 2A). Being closer to the LC+ time to onset was correlated with higher entorhinal FTP (r=0.34, p<0.001), but not with neocortical PiB (r=0.09, p=0.42; Figure 1B‐C). Moving closer to the time to onset to LC+ was associated with faster accumulation of entorhinal FTP (r=0.40, p<0.001), and faster cognitive decline (r=‐0.39, p<0.001; Figure 1D‐E). The LC+/EC+ onset trajectories were positively correlated (r=0.23, p=0.025; Figure 2B), and 36% of the individuals was LC‐EC‐, 3% LC‐EC+, 50% LC+EC‐ and 36% LC+EC+, and the time between LC+EC‐ to LC+EC+ was 6.06 years (SD:3.15; Figure 2C).

**Conclusions:**

LC integrity imaging allows for temporal modeling and our data suggests that LC integrity precedes accumulation of entorhinal tau by on average 6 years. Future work will evaluate the impact of risk and protective factors on these trajectories.